# 3D Flower-like Tin Monosulfide/Carbon Nanocomposite Anodes for Sodium-Ion Batteries

**DOI:** 10.3390/nano12081351

**Published:** 2022-04-14

**Authors:** Changju Chae, Sunho Jeong

**Affiliations:** 1 Division of Advanced Materials, Korea Research Institute of Chemical Technology (KRICT), Daejeon 34114, Korea; changju@krict.re.kr; 2 Department of Advanced Materials Engineering for Information and Electronics, and Integrated Education Institute for Frontier Science & Technology (BK21 Four), Kyung Hee University, Yongin-si 17104, Korea

**Keywords:** tin monosulfide, nanocomposite, anode, sodium, battery

## Abstract

The nanostructured tin monosulfide/carbon composites were synthesized by a simple wet chemical synthesis approach. It was revealed that the 3D flower-like tin monosulfide nanoparticles are usable as an active anode material for sodium-ion batteries, exhibiting a specific capacity of 480.4 mAh/g. The 3D flower-like tin monosulfide nanoparticles were wrapped with reduced graphene oxide sheets by a solvothermal heterogeneous synthetic method. By incorporating the reduced graphene oxide sheets as a mechanically flexible and electrically conductive additive, a specific capacity of 633.2 mAh/g was obtained from tin monosulfide/carbon nanocomposite anodes, providing an excellent rate capability even at a high current density condition of 5000 mA/g.

## 1. Introduction

In current battery technology, lithium-ion batteries are the most widely used energy storage unit for all types of portable electronics devices. With growing energy demand in recent decades, the market value for lithium-ion batteries has grown rapidly, and the field has expanded to large-scale electric energy storage units such as renewable power stations and electric vehicles [1,2,3]. However, lithium-ion batteries, as a major player in the current battery industry, face economic obstacles in cost-effective automotive applications because of high ore processing and cell fabrication costs, and the poor resource availability of lithium [4,5,6]. In contrast, sodium-ion batteries are recognized as a viable alternative to low-cost battery technology owing to the high abundance of sodium, low processing cost, and relatively high redox potential (the E*°* values for Li/Li^+^ and for Na/Na^+^ are 3.05 and 2.71 V vs. SHE at 298 K, 1 M, and 1 atm, respectively).

However, a practical realization of sodium-ion batteries is still made demanding by the intrinsic impediments of low energy density and poor cyclability, which are associated with the fact that the ionic radius of the sodium ion is 55% larger than that of lithium ion [7]. The larger ionic radius of the sodium ion makes it difficult to improve the electrochemical reaction kinetics, even in layer-structured electrodes, and to alleviate the significant structural expansion of the host electrodes. It was reported that a sodium ion can be intercalated rapidly into graphite anodes through a co-intercalation reaction of solvated sodium ions [8,9,10]; however, in certain types of anodes, a large ionic radius of the sodium ion results in an undesirable electroplating reaction on the surface of active materials [11].

Among various candidate anodes for sodium-ion batteries, ultrathin monosulfide (SnS) materials have the distinct advantages of high capacity, high rate capability, low hazard and toxicity, and excellent cost-effectiveness of raw materials. Tin monosulfide exhibits a theoretical capacity of 1022 mAh/g with conversion electrochemical reactions, whereas tin metal has a theoretical capacity of 847 mAh/g with the formation of a sodium-rich phase, Na_15_Sn_4_ [12]. To date, nanostructured SnS materials, including nanoparticles, nanowires, and nanosheets, have been studied to improve the electrochemical performance of SnS-based anodes [13,14,15,16,17,18,19,20]. Critical issues have been resolved to some degree by shortening the sodium ion and electron diffusion pathways, enhancing the electrode/electrolyte interfacial electrochemical reactions and circumventing microstructural changes during repeated charging/discharging reactions. However, even in such nanomaterial-based approaches, the electrochemical performance of SnS anodes is still limited by their poor electrical conductivity. To date, the methodologies for improving the electrical conductivity of SnS anodes have been suggested based on the spray pyrolysis synthesis [13], high-energy mechanical milling [14], carbonization [16,21], and CVD growth reaction [22]. These approaches involve multi-step synthetic procedures under the provision of high mechanical/thermal energy. To facilitate high-performance SnS-based anodes through a simple synthetic pathway, the development of a chemical strategy is necessary for improving the electrical conductance of the SnS anodes as well as maintaining their characteristic nanostructured morphological features.

In this study, we propose a chemical synthetic method of wrapping nanostructured SnS active materials with conductive carbon sheets. By designing heterogenous nucleation/growth reactions on top of the graphene oxide sheets at an elevated temperature of 200 °C, the nanostructured SnS particles were grown on thermally converted, relatively conductive reduced graphene oxide sheets without further annealing procedures. By establishing the carbon-wrapped SnS nanomaterials via a well-designed synthetic route, a specific capacitance of 633.2 mAh/g was obtained at a current density of 50 mA/g, and a capacitance of 66.6 mAh/g was maintained at a high current density condition of 5000 mA/g.

## 2. Materials and Methods

### 2.1. Synthesis of 3D Flower-like SnS Nanoparticles

In a 3-neck round flask, 71.8 g of oleylamine (70%, Simga Aldrich, St. Louis, MO, USA) and 4.2 g of oleic acid (90%, Simga Aldrich, St. Louis, MO, USA) were mixed and stirred. Then, 2.7 g of tin 2-ethylhexanoate (95%, Simga Aldrich, St. Louis, MO, USA) and 0.4 g of sulfur (99.98%, Simga Aldrich, St. Louis, MO, USA) were added to the mixture of oleylamine and oleic acid. The flask was connected to the Schlenk line and filled with Ar gas. Phenylhydrazine (97%, Simga Aldrich, St. Louis, MO, USA) was injected at a rate of 2 mL/min, and the reaction solution was heated to a temperature of 240 °C. After proceeding with the reactions for 1 h, the solution was allowed to cool to room temperature. The synthesized SnS particles were collected and washed in air with toluene using a centrifugation method. Chemical reactions were carried out under inert atmosphere, but all procedures were performed in air after cooling the reaction solutions to room temperature.

### 2.2. Synthesis of 3D Flower-like SnS/RGO Nanocomposites

Graphene oxides (GOs) were prepared by subsequent reactions of oxidation/exfoliation, following a modified Hummer’s method. Five grams of natural graphite flakes (Simga Aldrich, St. Louis, MO, USA) were mixed with 3.75 g of NaNO_3_ in a 2 L round-bottom flask containing 375 mL of H_2_SO_4_ (95%, Simga Aldrich, St. Louis, MO, USA) in an ice bath. 22.5 g of KMnO_4_ was added slowly, while the reaction temperature was kept below 20 °C. Then, the flask was placed in an oil bath at 30 °C, and it was removed from the oil bath 2 days later. After air cooling, 700 mL of 5 wt% H_2_SO_4_ was slowly added to the flask, and the reaction solution was stirred for 2 h. Next, 15 mL of 30 wt% H_2_O_2_ was slowly added. The color of the suspension changed from dark brown to yellow, and the reaction solution was stirred for 2 h. The as-obtained graphite oxides were purified with distilled water several times by centrifugation. Graphene oxide (GO) sheets were exfoliated by ultrasonication and redispersed in distilled water.

The solvent in the GO solution was exchanged with methyl alcohol, and 1.7 g of GO was mixed with 71.8 g of oleylamine, 1.0 g of oleic acid, 0.68 g of tin 2-ethylhexanoate, and 0.1 g of sulfur in the 3-neck round-bottom flask. The compositional ratio of carbon to SnS was set to be 2/8. The flask was connected to the Schlenk line and filled with Ar gas. Phenylhydrazine (97%, Simga Aldrich, St. Louis, MO, USA) was injected at a rate of 2 mL/min, and the reaction solution was heated to a temperature of 200 °C. After completing the reaction for 1 h, the solution was allowed to cool to room temperature. The synthesized SnS/RGO nanocomposites were collected and washed in air with toluene using a centrifugation method. The chemical reactions were carried out under inert atmosphere, but all procedures were performed in air after cooling the reaction solutions to room temperature.

### 2.3. Fabrication of Anodes for Sodium-Ion Batteries

Electrochemical tests were conducted using CR2032 coin cells. The working electrodes were prepared by casting a paste onto a current collector. The paste was composed of active materials, Super-P carbon black, and binder (active materials/Super-P/binder = 7:1.5:1.5 by weight). All working electrodes were pressed and vacuum-dried at 120 °C for 12 h. The loading mass was 1.5 mg/cm^2^. The 1 M NaClO_4_ with 5 wt% fluoroethylene carbonate in an ethylene carbonate/propylene carbonate (EC/PC) mixture (1:1 v/v) was used as an electrolyte. The volume of electrolyte was 40 microliters, and a glass fiber separator (GF/C, Whatman, Maidstone, UK) was used in this study. The half cells were assembled in an Ar-filled glovebox. The galvanostatic charge–discharge profile, cycling performance, and rate performance were investigated using battery testing equipment (TOSCAT-3100, Toyo Co., Ltd. Nagano, Japan). The first formation cycle was measured at a current density of 50 mA/g, and other cycles were measured at designated current density conditions.

### 2.4. Characterizations

Morphologies of synthesized SnS nanoparticles and SnS/RGO nanocomposites were observed by scanning electron microscopy (SEM, JSM-6700, JEOL, Tokyo, Japan) and transmission electron spectroscopy (TEM, FE-TEM, Tecnai G2 F30 S-Twin, FEI, Lausanne, Switzerland). The crystal structures were analyzed using an X-ray diffractometer (XRD, D/MAX-2200 V, Rigaku, Tokyo, Japan). The chemical structural analysis was performed by X-ray photoelectron spectroscopy (XPS, K-Alpha, Thermo Fisher Scientific, Waltham, MA, United States).

## 3. Results and Discussion

Three-dimensional nanostructured SnS nanoparticles were synthesized by a solvothermal reaction using tin 2-ethylhexanoate and elemental sulfur as source precursors. The oleylamine and oleic acid were used as a coordinating solvent and a capping molecule, respectively, to completely dissolve the sulfur powder and inhibit undesirable excessive growth reactions. After all reaction ingredients were mixed homogeneously under an inert atmosphere, phenylhydrazine as a reducing agent was injected to trigger the formation of the ionic compound at elevated temperatures. To exceed the supersaturation level for initiating the nucleation reaction, the chemical reactions were carried out at temperatures as high as 240 °C (lower than the boiling point of oleylamine). The nuclei larger than the critical radius underwent successive growth reactions as long as the solute concentration was maintained over the threshold limit for growth reactions [23]. In this regard, we incorporated a sufficient amount of reducing agent and set the reaction temperature at 240 °C.

**Figure 1:**
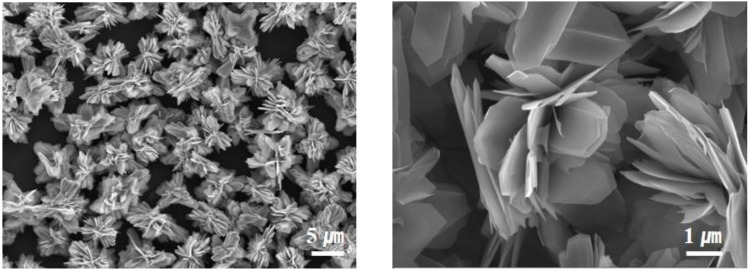
SEM images of F-SnS NPs synthesized in this study.

The SnS crystals tended to grow in a specific plane direction, reducing the interfacial surface energy among primary nanoparticles. For the case of the orthorhombic SnS crystal structure, the (100) facets were the most stable, owing to the strongest ionic interactions. Thus, it can be predicted that the SnS particles are grown, exposing the (100) facets. This two-dimensional growth behavior leads to the formation of the designated nanostructure in which ultrathin nano-sheets are assembled in a single individual particle [24]. As can be seen in Figure 1, the SnS nanoparticles synthesized in this study show a characteristic morphological feature in which two-dimensional petal-like building blocks, with smooth surface topology, are assembled one another at the center of the particle, resulting in the formation of 3D flower-like nanostructured particles. The internally interconnected nanostructure of the nano-petals suppresses undesirable aggregation of lamella geometry and rather maintains the hierarchical architecture with a high aspect ratio. Notably, the individual 3D flower-like SnS nanoparticle was constructed with 20~50 nm thick ultrathin petal sheets. This distinct morphological feature endows beneficial advantages as an anode material for sodium-ion batteries: (i) fast Na^+^ diffusion kinetics toward internal parts of active material, (ii) relaxed pulverization during repeated charging/discharging steps, (iii) highly efficient charge transfer from ultrathin SnS layer to neighboring conductive carbon particles, and (iv) high surface area to improve wettability of liquid electrolyte.

**Figure 2:**
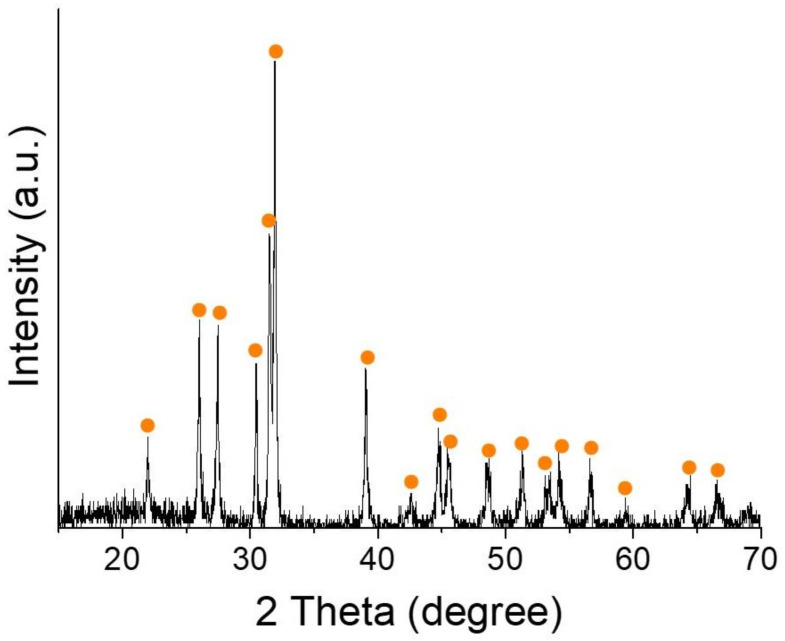
XRD result of the F-SnS NPs synthesized in this study. The dots indicate peaks associated with the SnS crystalline phase (herzenbergite, JCDPS 01-073-1859).

**Figure 3:**
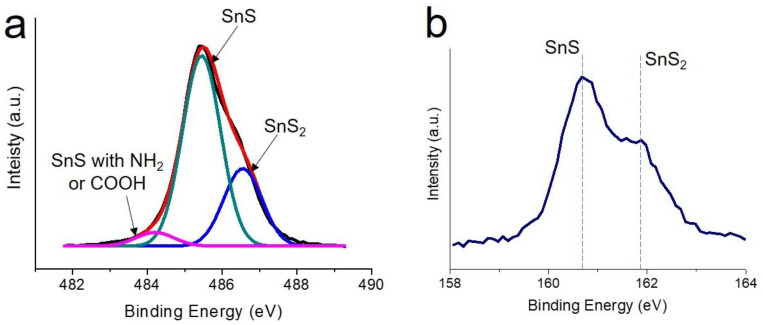
X-ray photoelectron (**a**) Sn 3d and (**b**) S 2p spectra for the F-SnS NPs.

Figure 2 shows the X-ray diffraction result for the 3D flower-like SnS nanoparticles (F-SnS NPs). It can be clearly observed that all peaks fit well to the crystalline structure of the SnS phase without leaving behind any kinds of impurities. The stoichiometric tin–sulfur ceramics, thermodynamically stable in ambient conditions, are the SnS and SnS_2_ phases. In this study, we incorporated a reducing agent to synthesize SnS nanoparticles. This led to chemical reduction reactions in both precursors. The formation of a low valence-tin cation, Sn^2+^, becomes more favorable, which stabilizes the synthesis reaction of the SnS phase rather than one in the SnS_2_ phase. To obtain more accurate information on the crystalline structure, we investigated by X-ray photoelectron spectroscopy (XPS) the Sn 3d and S 2p spectra for the F-SnS NPs. As can be seen in Figure 3a, the majority peak, positioned at 485.5 eV, was attributed to Sn^2+^ in the SnS phase. The peaks located at 484.2 and 486.6 eV resulted from Sn^2+^ in the SnS phase interacting with the amine (of oleylamine) and carboxyl group (of oleic acid), and the Sn^4+^ in SnS_2_ phase, respectively. Note that the XPS analysis is limited to the top surface. Thus, it can be speculated that the SnS crystalline phase forms preferentially, and the top surfaces of the petal sheets are partly composed of an SnS_2_ crystalline phase. It is also presumed that the surfaces of petal sheets are passivated chemically by solvent (oleyl amine) and organic capping (oleic acid) molecules. Because of the formation of this chemically driven surface passivation layer, the F-SnS NPs are stable in air without further passivation procedures. The coexistence of the SnS and SnS_2_ phases was also observed in the S 2p spectrum. The peaks positioned at 106.7 and 161.9 eV resulted from the S^2+^ of SnS and the S^4+^ of SnS_2_ phases, respectively.

**Figure 4:**
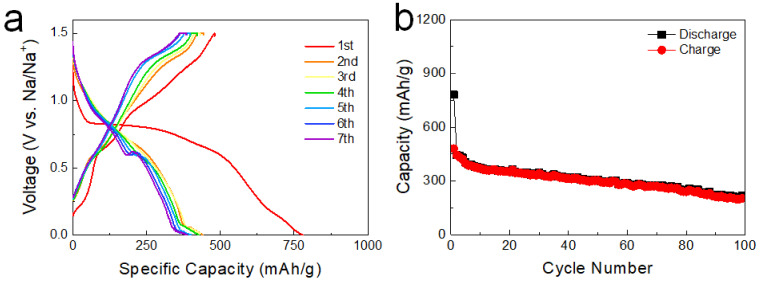
(**a**) Voltage profiles and (**b**) cyclability of the cells employing F-SnS NPs active materials. The current density was 50 mA/g.

Figure 4a shows the voltage profiles of the anode employing the F-SnS NPs active materials. After an irreversible electrochemical reaction in the first discharging step, a specific capacity approaching a value of 480.4 mAh/g was obtained at a current density of 50 mA/g. During the initial sodiation reaction, a long plateau was observed at 0.83 V. Then, the plateau was divided into two regions at 0.65 and 0.94 V in subsequent cycles. As for the cyclic voltammetry (CV) data of the F-SnS NPs anode cell, an intense peak appeared in the first reduction cycle and, in subsequent cycles, the peak observed at the first cycle was replaced with two separate peaks at lower and higher potentials (Figure S1). This activation reactions are, in general, observed in conversion reaction-based materials, which is attributable to a dramatic decrease in the domain size [25,26,27]. As can be seen in Figure 4b, the specific capacity was maintained without a dramatic retention of capacity during the 100-iteration cycling test. The specific capacity decreased gradually to the value of 216.7 mAh/g after the 100th cycling test. The limited capacity value was attributable to the low electrical conductivity of the F-SnS NPs and the inferior interfacial contact with conductive carbon particles. To resolve these issues, we designed carbon-wrapped F-SnS nanocomposites. We chose the graphene oxide (GO) sheets as a conductive wrapping additive to compensate for the poor electrical conductivity of the F-SnS NPs. As is well known, the GO sheets are not dispersed even in coordinating solvents [28]. We incorporated the graphene oxide (GO) sheets that can be dispersed in the reaction solution. The insulating GO sheets were converted into conductive RGO sheets with heterogeneous nucleation/growth reactions of F-SnS nanoparticles at a synthetic reaction temperature of 200 °C. In general, a thermal reduction of GO sheets can be triggered at temperatures over 200 °C [29].

**Figure 5:**
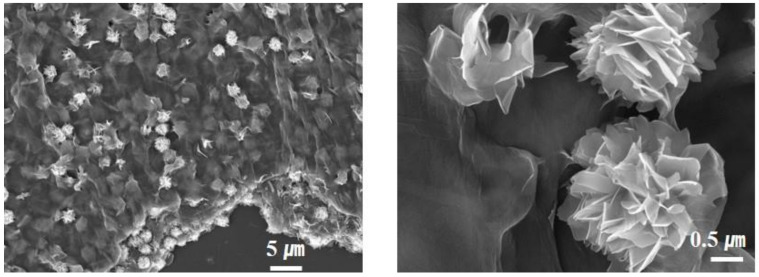
SEM images of the RGO/F-SnS NCs synthesized in this study.

We controlled the synthetic variables to induce a heterogeneous chemical reaction on top of the carbon sheets. It was achieved by regulating the reaction temperature and the amount of reducing agent to circumvent homogeneous nucleation/growth reactions. The heterogeneous chemical reactions were triggered predominantly on top of the layer with a high surface energy, because the activation energy for nucleation reactions decreases proportionally as a function of a contact angle on the foreign surface layer. The graphene oxide sheets have a number of functional groups that increase the surface energy and, in turn, decrease the contact angle. In fact, as can be seen in Figure 5, the F-SnS NPs were synthesized uniformly on carbon sheets without a formation of agglomerates. It can be clearly observed that the petal sheets of the F-SnS NPs were wrapped conformally by the RGO sheets. This can provide conductive pathways to outer conductive particles (carbon blacks in this study) in the anode layer. The RGO-wrapped F-SnS nanocomposites are denoted as RGO/F-SnS NCs in this study.

**Figure 6:**
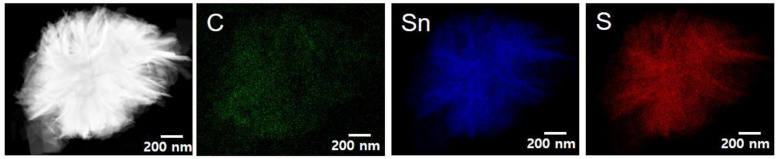
TEM elemental mapping images of the RGO/F-SnS NCs synthesized in this study.

**Figure 7:**
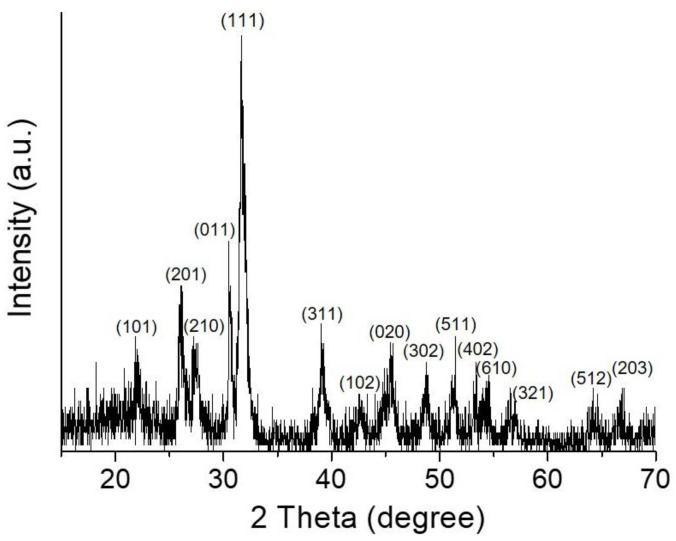
XRD results of the RGO/F-SnS NCs synthesized in this study.

**Figure 8:**
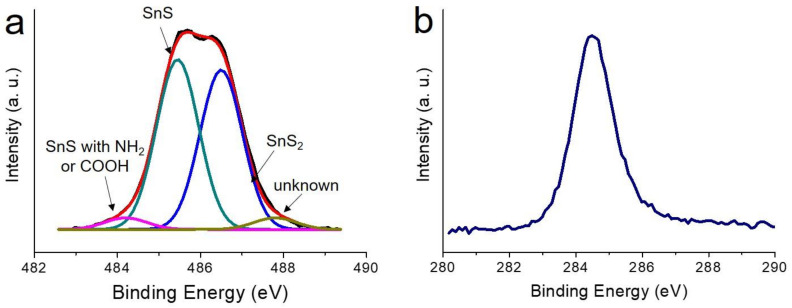
X-ray photoelectron (**a**) Sn 2d and (**b**) C 1s spectra for the RGO/F-SnS NCs.

**Figure 9:**
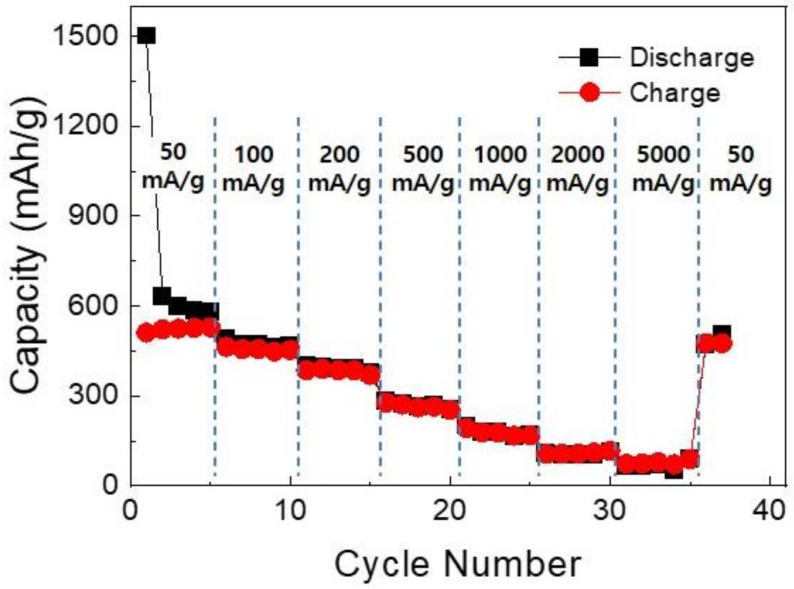
Rate capability at different current densities for the cell employing the RGO/F-SnS NCs active materials.

Figure 6 shows TEM elemental mapping results for the RGO/F-SnS NCs. It appears that the carbon, tin, and sulfur elements were distributed uniformly in the overall RGO/F-SnS NCs, indicative of the construction of well-designed carbon/SnS composite materials. As can be seen in Figure 7, the RGO/F-SnS NCs comprise a phase-pure SnS crystalline phase. Compared with the XRD result for the F-SnS NPs, a broad peak below 25° was observed in the XRD result for the RGO/F-SnS NCs. This peak was associated with the formation of well-stacked RGO sheets. Figure 8 shows the XPS spectra of the RGO/F-SnS NCs. In the XPS Sn 3D spectrum (Figure 8a), the peaks positioned at 485.5, 486.5, and 484.0 eV resulted from the Sn^2+^ in the SnS phase, Sn^4+^ in the SnS_2_ phase, and Sn^2+^ in the SnS phase interacting with the amine and carboxyl groups. The characteristics of the surficial chemical structure of RGO/F-SnS NCs were not different from those of the F-SnS NPs. This indicates that the synthetic method of inducing heterogenous chemical reactions on top of the carbon sheets is well-designed, maintaining the chemical/physical properties of F-SnS NPs. It is also confirmed that the GO sheets are completely converted into RGO sheets, without leaving behind surficial functional groups. As can be seen in Figure 8b, the peak from the C–C bond was observed at a binding energy of 284.5 eV, and other peaks originating from the functional groups were annihilated. Figure 9 shows the rate capabilities at different current density conditions for the RGO/F-SnS NC anode cell. The RGO/F-SnS NC anode cell exhibited a capacity as high as 633.2 mAh/g, after the first irreversible electrochemical reaction by surficial defects of RGO sheets. As for the CV data and discharge voltage profiles, it appears that the irreversible electrochemical reaction that occurred in the initial cycle and the electrochemical reactions after the 2nd cycle were almost identical to those of the F-SnS NP anode cell (Figures S2 and S3). For the RGO/F-SnS NC anode cell, the coulombic efficiency improved rapidly up to a value over 96% after the 2nd cycle (Table S1). It was confirmed that the RGO/F-SnS NC anode cell operated stably at a current density of 200 mA/g during the 80-time cycling test, without a noticeable change in the electrochemical impedance spectroscopy data (Figure S4). Notably, the RGO/F-SnS NC anode cell operated stably even at a high current condition of 5000 mA/g, showing a specific capacity of 66.6 mAh/g. Compared with the capacity value (at a current density of 50 mA/g) of the F-SnS NP anode cell, the specific capacity of the RGO/F-SnS NC anode cell increased by a factor of 1.3, from 480.4 to 633.2 mAh/g. This huge increment in capacity and the stable operation even at high current density conditions are attributable to the establishment of nanostructures in which active F-SnS NPs were wrapped uniformly by the relatively conductive, flexible carbon sheets.

## 4. Conclusions

In summary, we suggest the chemical synthetic method of synthesizing 3D flower-like SnS nanoparticles and SnS/carbon nanocomposites usable as anodes for sodium-ion batteries. The 3D flower-like SnS nanoparticles were synthesized by solvothermal reactions of tin and sulfur precursors in the presence of coordinating solvent and organic capping molecules. The SnS/carbon nanocomposites were synthesized by inducing heterogeneous synthesis reactions on top of graphene oxide sheets, by which 3D flower-like SnS nanoparticles were wrapped uniformly with thermally reduced graphene oxide sheets. It was found that by a facile formation of nanostructured active materials, a specific capacity of 633.2 mAh/g was obtained at a current density of 50 mA/g, and a specific capacity of 66.6 mAh/g was maintained at a high current density of 5000 mA/g.

## Data Availability

Data is contained within the article and Supplementary Materials.

## Supplementary Materials

The following supporting information can be downloaded at: https://www.mdpi.com/article/10.3390/nano12081351/s1. Figure S1. Cyclic voltammetry data for the cell employing the F-SnS NPs active materials. Figure S2. Cyclic voltammetry data for the cell employing the RGO/F-SnS NCs active materials. Figure S3. Voltage profiles for the cell employing the RGO/F-SnS NCs active materials. The current density was 200 mA/g. Figure S4. (a) Cyclability and (b) electrochemical impedance spectroscopy data during long-term cycling test for the cell employing the RGO/F-SnS NCs active materials. The current density was 200 mA/g. Table S1. The values in coulombic efficiency for the cell employing RGO/F-SnS NCs active materials.
