# Supplementary material for: 3D Flower-like Tin Monosulfide/Carbon Nanocomposite Anodes for Sodium-Ion Batteries
Source: Nanomaterials (Basel). 2022 Apr 14;12(8):1351. doi: 10.3390/nano12081351 (PMC9028553; doi:10.3390/nano12081351)
Supplement: Supplementary file 1 [file nanomaterials-12-01351-s001.zip › nanomaterials-1637616-supplementary.pdf]

nanomaterials

Supplementary Materials

3D Flower-like Tin Monosulfide/Carbon Nanocomposite Anodes for Sodium-Ion Batteries

Changju Chae [1] and Sunho Jeong [2,*]

[1] Division of Advanced Materials, Korea Research Institute of Chemical Technology (KRICT), Daejeon 34114, Korea; changju@krict.re.kr
[2] Department of Advanced Materials Engineering for Information and Electronics, Integrated Education Institute for Frontier Science & Technology (BK21 Four), Kyung Hee University, Yongin-si, 17104, Korea
* Correspondence: sjeong@khu.ac.kr

Table S1. The values in coulombic efficiency for the cell employing RGO/F-SnS NCs active materi-als.

Cycle #	Coulombic Efficiency
1st	32.1%
2nd	101.5%
3rd	94.1%
4th	94.9%
5th	95.5%
6th	95.9%
7th	96.0%

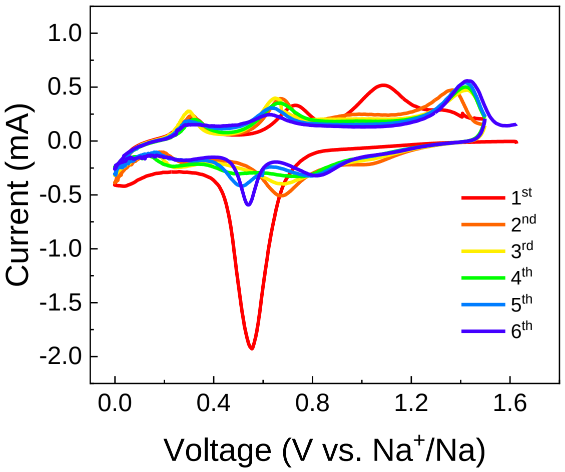

Figure S1. Cyclic voltammetry data for the cell employing the F-SnS NPs active materials.

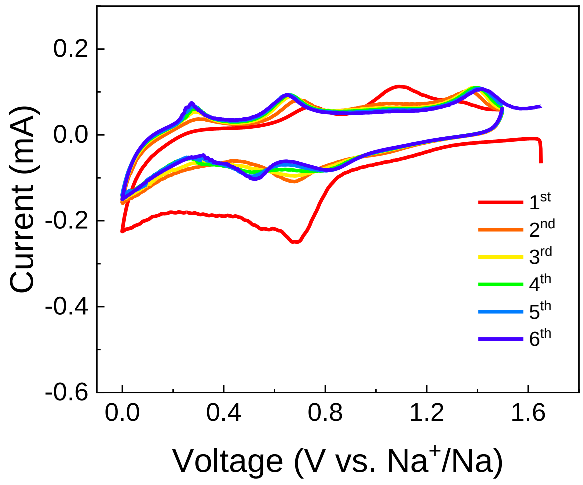

Figure S2. Cyclic voltammetry data for the cell employing the RGO/F-SnS NCs active materials.

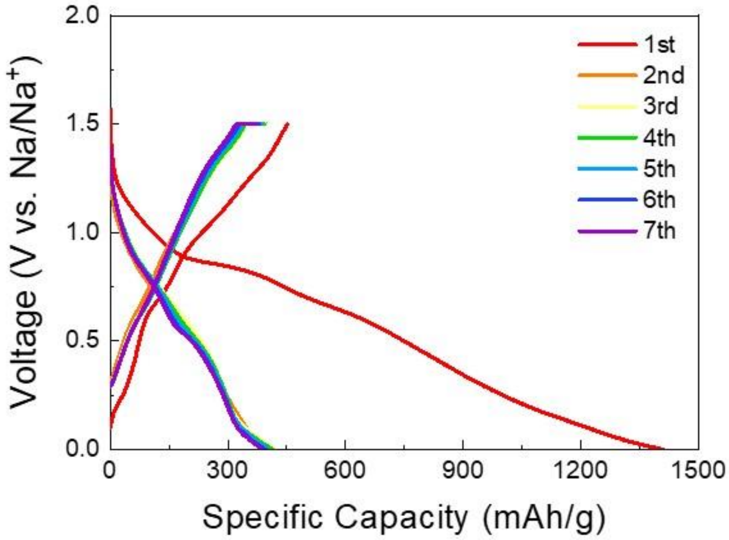

Figure S3. Voltage profiles for the cell employing the RGO/F-SnS NCs active materials. The current density was 200 mA/g.

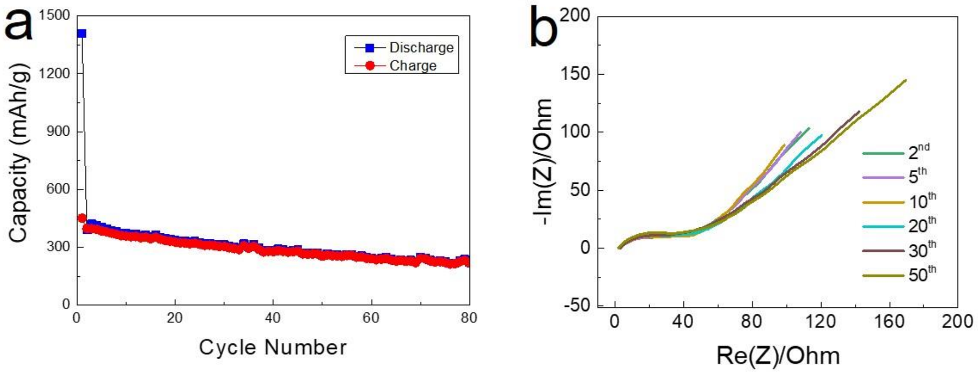

Figure S4. (**a**) Cyclability and (**b**) electrochemical impedance spectroscopy data during long-term cycling test for the cell employing the RGO/F-SnS NCs active materials. The current density was 200 mA/g.
